# RACK1 antagonizes TNF-α-induced cell death by promoting p38 activation

**DOI:** 10.1038/srep14298

**Published:** 2015-09-18

**Authors:** Qingyang Wang, Silei Zhou, Jing-Yang Wang, Junxia Cao, Xueying Zhang, Jing Wang, Kun Han, Qianqian Cheng, Guihua Qiu, Yawei Zhao, Xinying Li, Chunxia Qiao, Yan Li, Chunmei Hou, Jiyan Zhang

**Affiliations:** 1 Department of Molecular Immunology, Institute of Basic Medical Sciences, 27 Taiping Road, Beijing 100850, P. R. China

## Abstract

p38 mitogen-activated protein kinase (MAPK) activity has been reported to either promote or suppress cell death, which depends on cell type and stimulus. Our previous report indicates that p38 exerts a protective role in tumor necrosis factor (TNF)-α-induced cell death in L929 fibroblastoma cells. However, key molecules regulating p38 activation remain unclear. Here, we show that ectopic expression of scaffold protein receptor for activated C kinase 1 (RACK1) suppressed TNF-α-induced cell death in L929 cells, which was associated with enhanced p38 activation. Knockdown of endogenous RACK1 expression exhibited opposite effects. The protective role of RACK1 in TNF-α-induced cell death diminished upon blockade of p38 activation. Therefore, RACK1 antagonizes TNF-α-induced cell death through, at least partially, augmenting p38 activation. Further exploration revealed that RACK1 directly bound to MKK3/6 and enhanced the kinase activity of MKK3/6 without affecting MKK3/6 phosphorylation. Similar effects of RACK1 were also observed in primary murine hepatocytes, another cell type sensitive to TNF-α-induced cell death. Taken together, our data suggest that RACK1 is a key factor involved in p38 activation as well as TNF-α-induced cell death.

Tumor necrosis factor-α (TNF-α)-induced cell death contributes to tissue homeostasis, in which both p38 and c-Jun N-terminal protein kinase (JNK) are involved. p38 and JNK are members of the mitogen-activated protein kinase (MAPK) superfamily. The activation of p38 and JNK is typically mediated through sequential protein phosphorylation: MAPK kinase kinase (MAP3K or MEKK) → MAPK kinase (MAP2K or MKK) → MAPK, in response to multiple extracellular stimuli such as TNF-α^1^,^2^. MKK3 and MKK6 are the principal MAP2Ks responsible for the dual phosphorylation of p38 in the classical activation pathway^1^,^2^. JNK has been shown to contribute to TNF-α-induced cell death, whereas p38 activation antagonizes it^3^. However, key molecules regulating p38 activation remain unclear.

Receptor for activated C kinase 1 (RACK1) was originally identified on the basis of its ability to anchor activated form of protein kinase C (PKC) and is now recognized as a multi-functional scaffold protein^4^,^5^. It has been reported that RACK1 can associate with both PKC and JNK, which enables PKC to phosphorylate JNK at Ser129 and thereby facilitates the basal and inducible dual phosphorylation of JNK by MKK4/7 in human melanoma cells^6^,^7^. However, the interaction of RACK1 with JNK was not detected by another group in COS7 African green monkey kidney cells^8^. Instead, the binding of RACK1 to MEKK4 has been revealed to be essential, but not sufficient, for MEKK4-mediated JNK activation in this cell model^8^. In addition, our previous study indicates that RACK1 enhances JNK activation by directly binding to and facilitating the interaction between MKK7 and upstream MAP3Ks in human hepatocellular carcinoma cells^9^. Thus, the molecular mechanism by which RACK1 regulates the JNK pathway may be cell context-dependent. Despite of such findings, it remains unknown whether RACK1 regulates p38 activation.

L929 fibroblastoma cells are sensitive to TNF-α-induced cell death^3^,^10^. In this study, we report that RACK1 augments p38 activity and thereby promotes the survival of L929 cells by directly binding to MKK3/6 and enhancing MKK3/6 activity. We have also found the same effects of RACK1 in primary murine hepatocytes.

## Results

### RACK1 suppresses TNF-α-induced cell death in L929 fibroblastoma cells

**Figure 1:**
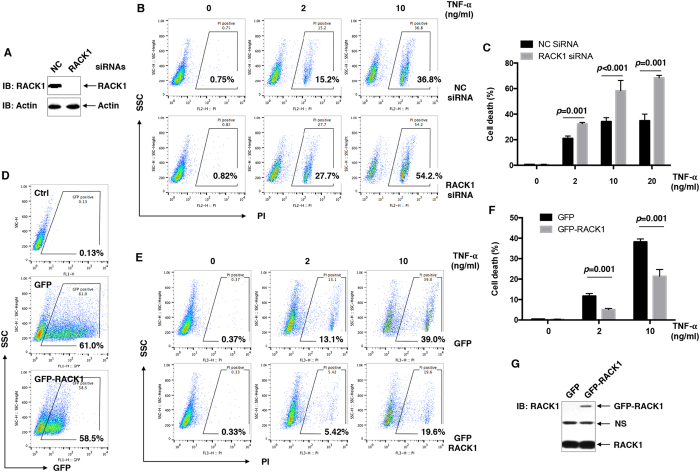
RACK1 suppresses TNF-α-induced cell death in L929 cells. (**A**) L929 cells were transfected with non-targeting control (NC) siRNA or RACK1 siRNA. 72 hours later, cell lysates were harvested and subjected to immunoblotting (IB) analysis with the indicated antibodies (Abs). (**B**,**C**) L929 cells were transfected with NC siRNA or RACK1 siRNA. 48 hours later, cells were treated with 0, 2, 10, or 20 ng/ml TNF-α for 24 hours. Cell death was measured by flow cytometry analysis of PI staining. Representative data (**B**) and statistical data (*C*, mean ± SD, n = 3) of three independent experiments are shown. *D*–*G*, L929 cells were transfected with mammalian expression vectors encoding GFP or GFP-RACK1. 48 hours later, cells were treated with 0, 2, or 10 ng/ml TNF-α for 24 hours followed by flow cytometry analysis of PI staining. GFP positive cells were gated (**D**) and analyzed. Representative data (**E**) and statistical data (**F**, mean ± SD, n = 3) of three independent experiments are shown. Ectopic expression of GFP-RACK1 was confirmed by IB analysis (**G**). NS, nonspecific.

Fibroblastoma cell line L929 is highly sensitive to TNF-α-induced cell death, and thereby is widely used to reveal the mechanisms underlying TNF-α-induced cell death^3^,^10^. Our previous study has demonstrated that TNF-α-induced cell death in L929 cells can be simply analyzed by propidium iodide (PI) staining^3^. To investigate whether RACK1 affects this process, we analyzed the effects of RACK1 loss-of-function or gain-of-function in L929 cells. L929 cells were transiently transfected with RACK1 small interfering RNA (siRNA) or non-targeting control (NC) siRNA by using Amaxa nucleofector II. 72 hours later, L929 cells were treated with various doses of TNF-α for 24 hours, followed by cell death assays with PI staining. Immunoblotting (IB) analysis confirmed the efficient knockdown of endogenous RACK1 (Fig. 1A), which led to increased cell death in TNF-α-treated L929 cells (Fig. 1B,C). By contrast, GFP-RACK1 ectopic expression exhibited opposite effects (Fig. 1E,F), when GFP positive cells were gated and analyzed (Fig. 1D). IB analysis confirmed the ectopic expression of GFP-RACK1 (Fig. 1G). Densitometric readings revealed that exogenous GFP-RACK1 relative to endogenous RACK1 was only about 13% (Fig. 1G), suggesting that slight increase of the total level of RACK1 protein is enough to protect against TNF-α-induced cell death in L929 cells.

### RACK1 suppresses TNF-α-induced cell death via promoting p38 activation

**Figure 2:**
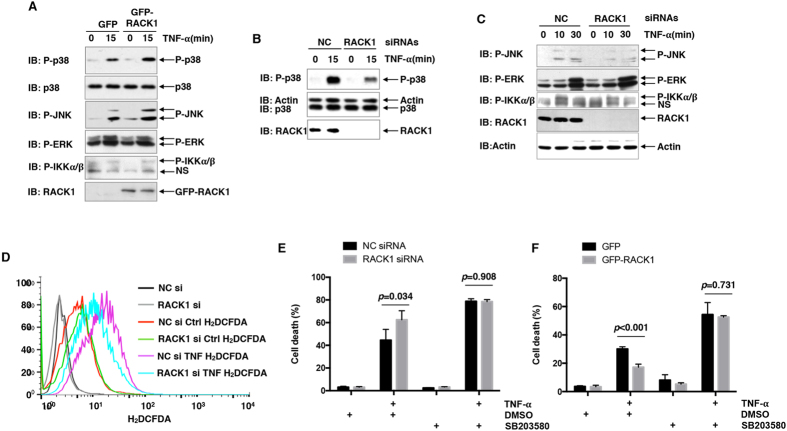
RACK1 suppresses TNF-α-induced cell death via promoting p38 activation. (**A–C**) L929 cells were transfected with mammalian expression vectors or siRNAs as indicated. After stimulation with 10 ng/ml TNF-α for various periods of time as indicated, cell lysates were harvested and subjected to IB analysis with the indicated Abs. (**D**) L929 cells were transfected with RACK1 siRNA or non-targeting control siRNA. 72 h later, cells were treated with or without TNF-α (10 ng/ml, 90 min). ROS generation was measured by incubating cells with carboxy-H_2_DCFDA, followed by flow cytometry. (**E**,**F**) L929 cells were transfected with siRNAs (**E**) or mammalian expression vectors (**F**) as indicated. After pretreatment with or without SB203580 (10 μM, 30 min), cells were stimulated with 10 ng/ml TNF-α for 24 hours or left untreated. Cell death was then measured by PI staining and statistical data of three independent experiments are shown by mean ± SD; n = 3.

Our previous data have shown that p38 plays a pro-survival role in TNF-α-induced cell death of L929 cells^3^. Whether RACK1 affects p38 activity and thereby affects TNF-α-induced cell death remains unknown. To investigate this issue, we over-expressed GFP or GFP-RACK1 in L929 cells, followed by 10 ng/ml TNF-α stimulation for 15 min. IB analysis revealed that TNF-α-induced dual phosphorylation of p38 (P-p38), which indicates p38 activation, was augmented upon GFP-RACK1 over-expression, as compared to GFP over-expression (Fig. 2A). However, basal p38 phosphorylation was not augmented upon GFP-RACK1 over-expression (Fig. 2A). Basal and TNF-α-induced dual phosphorylation of JNK (P-JNK), which indicates JNK activation^6^,^7^, was also augmented upon GFP-RACK1 over-expression, whereas that of IKKα/β and ERK (P-IKKα/β and P-ERK) remained unchanged (Fig. 2A). In line with these data, silencing of endogenous RACK1 in L929 cells resulted in obviously attenuated activation of p38 and JNK, but not that of IKKα/β and ERK, in response to TNF-α (Fig. 2B,C). Our previous study suggests JNK activity contributes to TNF-α-induced cell death of L929 cells^3^. JNK has been demonstrated to potentiate TNF-α-induced cell death by increasing the production of reactive oxygen species (ROS)^11^. Consistently, RACK1 knockdown in L929 cells led to reduced ROS generation in response to TNF-α treatment (Fig. 2D). Thus, it is unlikely that RACK1 suppresses TNF-α-induced cell death by enhancing JNK activation. To address whether RACK1 suppresses TNF-α-induced cell death by promoting p38 activation, p38 inhibitor SB203580 was used to block the activity of p38. PI staining revealed that pretreatment with SB203580 led to elevated cell death upon TNF-α stimulation (Fig. 2E,F). Furthermore, the detrimental effect of RACK1 knockdown and the protective effect of RACK1 over-expression on TNF-α-induced cell death diminished upon SB203580 pretreatment (Fig. 2E,F). Collectively, our data suggest that RACK1 suppresses TNF-α-induced cell death via promoting p38 activation.

### RACK1 interacts with MKK3 and MKK6 *in vivo* and *in vitro*

**Figure 3:**
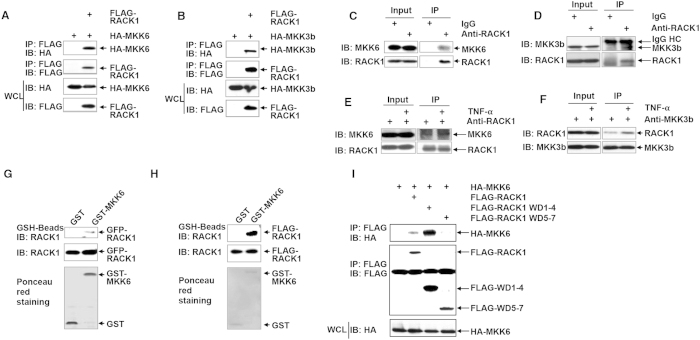
RACK1 interacts with MKK6 and MKK3b *in vitro* and *in vivo*. (**A**,**B**) 293T cells were transfected with various mammalian expression vectors as indicated. Cell lysates were subjected to immunoprecipitation (IP) with anti-FLAG Ab, followed by SDS-PAGE and IB analysis with the indicated Abs. (**C**,**D**) Cell lysates of L929 cells were subjected to IP with control IgG or anti-RACK1 Ab, followed by IB analysis with the indicated Abs. HC, heavy chain. (**E**,**F**) L929 cells were treated with or without 10 ng/ml TNF-α for 10 min. Cell lysates were subjected to IP with anti-RACK1 Ab or anti-MKK3b Ab, followed by IB analysis with the indicated Abs. (**G**,**H**) GST or GST-MKK6 bound to glutathione-Sepharose beads were incubated with lysates of 293T cells expressing GFP-RACK1 or *in vitro* translated FLAG-RACK1. Precipitates were subjected to SDS-PAGE and IB analysis with anti-RACK1 Ab. *I* 293T cells were transfected with various mammalian expression vectors as indicated. Cell lysates were subjected to IP with anti-FLAG Ab, followed by SDS-PAGE and IB analysis with the indicated Abs.

Since we previously confirmed that RACK1 could interact with MKK7^9^, it is possible that RACK1 interacts with MKK3 and MKK6. The *in vivo* interaction of RACK1 with MKK6 was confirmed by co-immunoprecipitation (co-IP) assays. HA-tagged MKK6 (HA-MKK6) and HA-MKK3b co-precipitated with FLAG-tagged RACK1 (FLAG-RACK1), respectively, in human 293T cells (Fig. 3A,B). Next, the endogenous interaction of RACK1 with MKK3/6 was confirmed by endogenous co-IP assays in L929 cells (Fig. 3C,D). Notably, the endogenous interaction between RACK1 and MKK3/6 enhanced upon TNF-α stimulation (Fig. 3E,F). To test whether RACK1 could directly bind to MKK6, *in vitro* glutathione S-transferase (GST)-pull down assays were performed. As expected, GFP-RACK1 in lysates of 293T cells was precipitated by GST-MKK6 but not by GST (Fig. 3G). A similar phenomenon was observed when using *in vitro* translated FLAG-RACK1 (Fig. 3H). RACK1 contains seven Trp-Asp (WD) repeats. Co-IP analysis revealed that HA-MKK6 co-precipitated with co-expressed RACK1 deletion mutant that included WD domains one to four (RACK1 WD1-4), but not with co-expressed RACK1 WD5-7 (Fig. 3I). Taken together, our data suggest that RACK1 could engage in a direct interaction with MKK3/6 *in vivo* and *in vitro.*

### Binding of RACK1 to MKK3/6 enhances the kinase activity of MKK3/6

**Figure 4:**
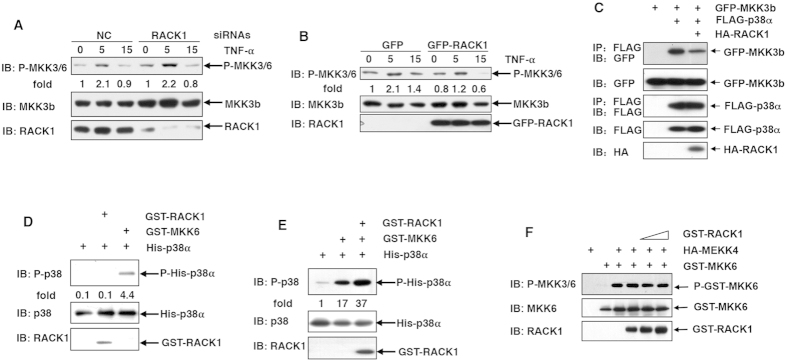
RACK1 enhances the kinase activity of MKK3/6. (**A**,**B**) L929 cells were transfected with various siRNAs (**A**) or mammalian expression vectors (**B**) as indicated. Cells were stimulated with 10 ng/ml TNF-α for various periods of time as indicated, followed by IB analysis with the indicated Abs. Densitometric readings are shown for P-MKK3/6 and normalized with MKK3b protein. (**C**) 293T cells were transfected with various mammalian expression vectors as indicated. Cell lysates were subjected to IP with anti-FLAG Ab, followed by SDS-PAGE and IB analysis with the indicated Abs. (**D**,**E**) GST-MKK6 and His-p38α were incubated with or without GST-RACK1 in kinase buffer at 30 °C for 1 hour. Nonradioactive ATP was added to drive the reaction. Samples were then subjected to IB analysis with the indicated Abs. Densitometric readings are shown for P-His-p38α and normalized with His-p38α protein. (**F**) Suboptimal amount of HA-MEKK4 was incubated with GST-MKK6 in the presence of various amount of GST-RACK1 in kinase buffer at 30 °C for 1 hour. Nonradioactive ATP was added to drive the reaction. Samples were then subjected to IB analysis with the indicated Abs.

MKK3 and MKK6 are the principal MAP2Ks responsible for the dual phosphorylation of p38 in response to various extracellular stimuli including TNF-α^1^,^2^. Now that RACK1 enhances p38 phosphorylation upon TNF-α stimulation, it is possible that RACK1 enhances TNF-α-induced MKK3/6 phosphorylation. To analyze this issue, we first examined whether RACK1 knockdown led to diminished MKK3/6 phosphorylation in response to TNF-α. To our surprise, TNF-α-induced MKK3/6 phosphorylation did not decrease upon silencing of endogenous RACK1 expression (Fig. 4A). Moreover, GFP-RACK1 over-expression did not enhance TNF-α-induced MKK3/6 phosphorylation, as compared to GFP over-expression (Fig. 4B). Next, we assumed that RACK1 could promote the interaction of p38α with its upstream kinases. Unexpectedly, RACK1 did not enhance the interaction of MKK3b with p38α but weakened it in 293T cells (Fig. 4C).

Scaffold proteins could enhance the kinase activity of a kinase without affecting its phosphorylation^4^. To test whether RACK1 enhances the kinase activity of MKK3/6, purified GST or GST-RACK1 mixed with His-p38α and GST-MKK6 were subjected to nonradioactive *in vitro* kinase assays (KA). IB analysis revealed that RACK1 enhanced the phosphorylation of His-p38α in the presence of GST-MKK6, while RACK1 alone was unable to promote the autophosphorylation of His-p38α (Fig. 4D,E). By the way, RACK1 had no effect on the phosphorylation of MKK6 by suboptimal amount of upstream kinase MEKK4 (Fig. 4F). Taken together, our data suggest that RACK1 promotes the phosphorylation of p38 by enhancing the kinase activity of MKK3/6.

### RACK1 plays a pro-survival role in TNF-α-induced cell death in primary murine hepatocytes

**Figure 5:**
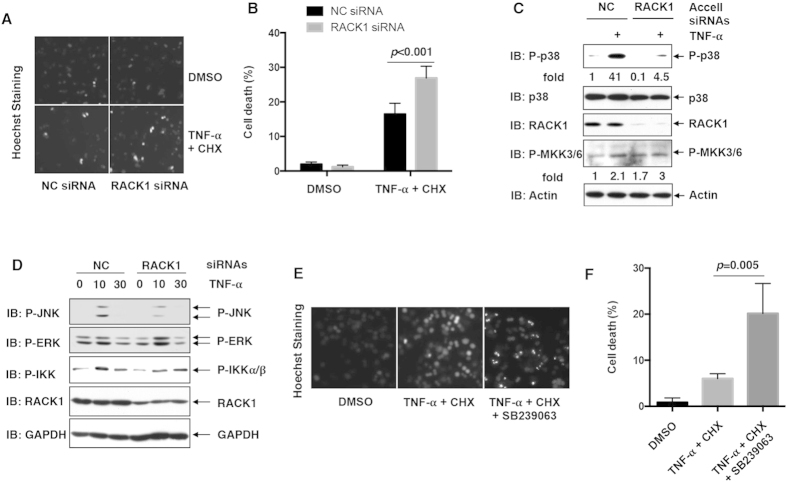
RACK1 affects TNF-α-induced cell death in primary murine hepatocytes. (**A**,**B**) Primary murine hepatocytes were transfected with NC siRNA or RACK1 siRNA by using Accell RNA delivery system. Cells were then treated with 5 μg/ml CHX and 10 ng/ml TNF-α for 24 hours. Cell death was determined by Hoechst staining. Representative data (**A**) and statistical data (**B**, mean ± SD, n = 3) of three independent experiments are shown. (**C**,**D**) Primary murine hepatocytes were transfected as described above. After stimulation with 10 ng/ml TNF-α for various periods of time as indicated, cell lysates were subjected to IB analysis with the indicated Abs. Densitometric readings are shown for P-p38 and P-MKK3/6 and normalized with Actin protein. (**E**,**F**) After pretreatment with SB239063 (10 μM, 30 min), primary murine hepatocytes were treated with 5 μg/ml CHX and 2 ng/ml TNF-α for 24 hours. Cell death was determined by hoechst staining. Representative data (**E**) and statistical data (**F**, mean ± SD, n = 3) of three independent experiments are shown.

In this work, we also proposed that the protective role of RACK1 against TNF-α-induced cell death was not limited to L929 cells. Hepatocytes, similar to L929 cells, were confirmed to be sensitive to TNF-α-induced cell death *in vivo*^12^. And *in vitro*, TNF-α induces cell death in primary murine hepatocytes in the presence of protein synthesis inhibitor cycloheximide (CHX)^12^. In this scenario, primary murine hepatocytes were isolated using a two-step collagenase perfusion technique from adult male mice at the age of 8 to 10 weeks. An Accell RNA delivery system was used for RNA interference in primary murine hepatocytes, followed by treatment with 10 ng/ml TNF-α plus 5 μg/ml CHX. Hoechst staining was used to measure cell death. As expected, RACK1 Accell siRNA augmented TNF-α-induced cell death (Fig. 5A,B). IB analysis confirmed that RACK1 was efficiently knocked down in primary murine hepatocytes (Fig. 5C). Moreover, RACK1 knockdown in primary murine hepatocytes led to decreased basal p38 phosphorylation as well as decreased TNF-α-induced p38 phosphorylation without inhibiting MKK3/6 phosphorylation (Fig. 5C). Similar to our previous data about RACK1 knockdown in L929 cells, IB analysis revealed that RACK1 knockdown in primary murine hepatocytes hindered TNF-α-induced JNK phosphorylation while marginally affecting IKKα/β and ERK phosphorylation (Fig. 5D). To further confirm the role of p38 in TNF-α-induced cell death of hepatocytes, SB239063, another p38 specific inhibitor, was used before TNF-α plus CHX treatment. Hoechst staining revealed that 2 ng/ml TNF-α plus 5 μg/ml CHX just caused slight cell death but cell death dramatically enhanced in the presence of SB239063 (Fig. 5E,F). Collectively, these data suggest that p38 activity plays a pivotal role in antagonizing TNF-α-induced cell death in hepatocytes and RACK1 exerts its pro-survival function by regulating p38 activity.

## Discussion

Scaffolding protein RACK1 has been reported to regulate various cellular processes including cell cycle progression, tumor development, and circadian clock^9^,^13^,^14^,^15^,^16^, but little is known about how RACK1 affects cell death. Here, we report that RACK1 suppresses TNF-α-induced cell death via promoting p38 activation. Furthermore, we have found that RACK1 enhances TNF-α-induced p38 phosphorylation by directly binding to its up-stream kinases MKK3/6 and enhancing MKK3/6 kinase activity. Although a previous study failed to observe the interaction between RACK1 and MKK6 in COS7 African green monkey kidney cells^8^, in our hands both HA-MKK6 and HA-MKK3b co-precipitated with co-expressed FLAG-RACK1 in 293T cells (Fig. 3A,B). More importantly, the physiological interaction of RACK1 with MKK3/6 in L929 cells was revealed by immunoprecipitating endogenous proteins (Fig. 3C,D). In addition, GST-pull down assays indicate the interaction is direct (Fig. 3G,H). In line with our observations, a recent study has demonstrated that RACK1 also mediates the cytokine RANKL (receptor activator of NF-κB ligand)-dependent activation of p38 by interacting with MKK6 in osteoclast precursors. Consequently, local administration of RACK1 siRNA into mice calvariae reduced the RANKL-induced bone loss through reducing the numbers of osteoclasts^17^. The reason(s) for the above discrepancy are still unknown. It is possible that the physiological binding of RACK1 to MKK3/6 depends on cell type and stimulus.

Notably, the endogenous interaction between RACK1 and MKK3/6 enhanced upon TNF-α stimulation (Fig. 3E,F). Enhanced MKK6/RACK1 interaction has also been reported to occur upon RANKL treatment and a dominant negative MKK6 mutant showed markedly decreased binding to RACK1 compared to that of wild-type MKK6^17^. Taken together, these facts suggest that MKK3/6 phosphorylation is important for their binding to RACK1. It is possible that some conformational changes resulting from MKK3/6 phosphorylation leads to the subsequent binding to RACK1. Consequently, RACK1 further promotes the activation of p38 pathway by enhancing MKK3/6 kinase activity. On the other hand, it is interesting that RACK1 only promotes TNF-α-induced p38 phosphorylation, but not basal p38 phosphorylation, in L929 cells (Fig. 2A,B), whereas it enhances both basal and TNF-α-induced p38 phosphorylation in primary murine hepatocytes (Fig. 5C). It is possible that basal p38 phosphorylation in L929 cells mainly comes from p38 autophosphorylation, whereas its counterpart in primary murine hepatocytes mainly depends on MKK3/6. Future studies are required to address this issue.

TNF-α is a pleiotropic cytokine that exerts its function by activating multiple signaling pathways including p38, JNK, ERK, and IKKα/β^12^. Even though some reports in the literature suggest that RACK1 might regulate the activation of ERK and IKK^18^,^19^, we failed to observe such a role in a previous study^9^. Instead, we have found that RACK1 enhances JNK activation by directly binding to MAP2K MKK7 in human hepatocellular carcinoma cells. Here, we again report that RACK1 enhances JNK activation while marginally affecting IKKα/β and ERK in L929 cells and primary murine hepatocytes. Furthermore, we disclose RACK1 enhances TNF-α-induced p38 activation by directly binding to MAP2K MKK3/6 in these cells. TNF-α-induced cell death is a common reason in a variety of forms of tissue injury such as liver damage^20^,^21^. JNK activity has been shown to contribute to TNF-α-induced cell death^3^,^22^,^23^. Even though RACK1 promotes TNF-α-induced JNK activation (Figs 2A,C and 5D), the enhancement of TNF-α-induced p38 activation by RACK1 obviously plays a predominant role in this process. Furthermore, this study suggests that normal p38 activity is very important to liver protection when the liver is undergoing severe inflammation. And this notion may explain why abnormally elevated liver enzymes in the serum could be frequently observed when p38 inhibitor is used to control severe inflammation in patients suffering sepsis^24^.

Taken together, our study reveals a novel mechanism underlying TNF-α-induced cell death: scaffolding protein RACK1 regulates the kinase activity of MKK3/6 and the phosphorylation of p38, thereby antagonizes TNF-α-induced cell death. By the way, due to the important pro-survival role of p38 in hepatocytes, the use of p38 inhibitor to control severe inflammation must be cautious.

## Methods

### Reagents

Antibodies against phosphorylated p38, phosphorylated MKK3/6, MKK6, and MKK3b were from Cell Signaling Technology (Beverly, MA, USA). Antibodies against p38, HA, GFP, and Actin were from Santa Cruz Biotechnology (Santa Cruz, CA, USA). Antibody against RACK1 was from BD Biosciences (Franklin Lakes, NJ, USA). Glutathione-Sepharose beads, Protein A-Sepharose beads, Hanks’ balanced salt solution (HBSS), collagenase I, PI, hoechst (H33258), cycloheximide, and antibody against FLAG were from Sigma-Aldrich (St. Louis, MO, USA). p38 inhibitors SB203580 and SB239063 were from CalBiochem (San Diego, CA, USA). ECL chemiluminescence kit was from Amersham (Arlington Heights, IL, USA). *In vitro* protein translation system was from Promega (Madison, WI, USA).

### Isolation and culture of primary murine hepatocytes

Male C57BL/6 mice at the age of 8 to 10 weeks were obtained from Beijing Vital River Laboratory Animal, Inc. (Beijing, China, http://www.vitalriver.com.cn). Mice were immediately used upon arrival. The treatment of mice in this study was in strict agreement with the guidelines set by the Institute of Basic Medical Sciences. All efforts were made to minimise the suffering of the mice. The number of mice used in this study was 10 and all mice were euthanized. The two-step collagenase perfusion technique was used for the isolation of large numbers of viable adult hepatocytes^21^. Briefly, after mice were anaesthetised by administrating pentobarbital i.p. at 50 mg/kg, skin was cut and vena cava and portal vein were exposed. Cold HBSS containing EDTA were perfused into liver through portal vein for 5 min, followed by the introduction of collagenase into liver for another 5 min. After perfusion, livers were removed and pressed through a 200-gauge stainless steel mesh. After washing twice with cold DMEM by centrifugation at 50 × *g* for 3 min, cells were re-suspended with 50% percoll and followed by centrifugation at 50 × g for 10 min. The supernatants were removed as much as possible and the cell pellets were washed for three times. The isolated hepatocytes were maintained in William’s E Medium with 5% fetal bovine serum, 100 U/ml penicillin and 100 μg/ml streptomycin.

### Cell culture and transfection

93T cells and L929 cells were grown in Dulbecco’s modified Eagle medium (DMEM) supplemented with 10% fetal bovine serum, 100 U/ml penicillin and 100 μg/ml streptomycin. Plasmids or siRNAs were transfected into 293T cells with lipofectamine 2000 (Invitrogen, Carlsbad, CA, USA) according to the manufacture’s protocol, whereas transfection of L929 cells was performed by using Amaxa nucleofector II (kit V, Lonza, Switzerland) according to the manufacture’s protocol. RNA interference in primary murine hepatocytes was carried out with Accell siRNA (Dharmacon, Lafayette, CO, USA) utilizing the Accell siRNA delivery protocol. Plasmids used in this work have been described previously^9^. Murine RACK1 siRNA (GGTCCAGGATGAGAGTCAT) and NC siRNA were from GenePharma (Shanghai, China).

### Flow cytometry

TNF-α-induced cell death of L929 cells could be simply analyzed by PI staining because the majority of L929 cells undergoing cell death were Annexin-V/PI double positive^3^. Briefly, cells were digested with 0.25% trypsin for about 2 min with gently shaking and were then harvested. After washing with PBS twice, cell pellet was resuspended in 200 μl PBS containing 5 μg/ml PI and incubated at dark for 5 min, followed by flow cytometry analysis.

### *In vitro* translation, GST-pull down and KA

GST, GST-MKK6, GST-RACK1, and His-p38α were expressed and purified as described previously^25^. *In vitro* translation of RACK1 was performed by using Promega protein expression system, according to the manufacture’s protocol. For GST-pull down assays, GST-MKK6, *in vitro* translated RACK1 and glutathione-Sepharose beads were mixed together and rotated at 4 °C for 3 hours. After extensive washing, samples were subjected to IB analysis. For KA, proteins were mixed and incubated at 30 °C for 1 hour in kinase buffer (20 mM HEPES, pH 7.6, 20 mM MgCl_2_, 1 mM DTT, 20 μM nonradioactive ATP). After reaction, samples were added with 4 × SDS sample buffer and heated at 95 °C for 5 min, and subjected to IB analysis.

### Co-IP and IB analysis

Co-IP and IB analysis were performed as described previously^9^. Briefly, for co-IP, cells were lysed and harvested in lysis buffer (20 mM Tris-Cl, PH 7.6, 120 mM NaCl, 10% Glycerol, 2 mM EDTA, 1% Triton X-100, 1 mM PMSF, 1 mM Na_3_VO_4_, 10 μg/ml aprotinin). After centrifugation, the supernatants were incubated with the indicated antibodies in the presence of 30 μl protein A-Sepharose beads at 4 °C for 4 hours. The precipitates were washed for 3 to 5 times depending on the scenarios. For IB analysis, cell lysates or KA/co-IP samples were subjected to SDS-PAGE, followed by transferring to nitrocellulose membranes. Nitrocellulose membranes were incubated with 5% (w/v) dry non-fat milk in washing buffer (20 mM Tris-Cl, PH 7.6, 150 mM NaCl, 0.1% Tween 20) at room temperature for 1 hour to block the nonspecific protein binding. Primary antibodies were diluted in washing buffer containing 3% (w/v) BSA and applied to the membranes for overnight at 4 °C. After extensive washing, the membranes were incubated with peroxidase-conjugated antibodies for 1 hour at room temperature and washed again. Immunoreactive bands were visualized with the ECL chemiluminescence kit.

### Hoechst staining

Primary hepatocytes were stained with hoechst (H33258) at 37 °C for 15 min and washed with PBS twice. Nuclear condensation and DNA fragmentation were visualized by fluorescence microscopy.

### ROS production assays

A LIVE Green Reactive Oxygen Species Detection Kit (Life Technologies, Eugene, OR, USA) was used for detecting the generation of ROS. Briefly, cells were incubated in serum-free RPMI medium containing 2 μM carboxy-H_2_DCFDA (Molecular Probes) at 37 °C for 30 min. Cells were washed with PBS and were immediately subjected to flow cytometry to analyse the intensity of green fluorescence at a 488 nm excitation wavelength.

### Statistical analysis

The data were shown as mean ± standard deviations (SD). Student’s *t* test was employed to determine significance between two groups and One Way Anova analysis was used to determine significance among several groups. Differences were considered statistically significant when *p *< 0.05.

### Ethics statement

All experimental protocols used in this work were approved by the institutional review board of the Institute of Basic Medical Sciences. The methods were carried out in accordance with the approved guidelines.

## Additional Information

**How to cite this article**: Wang, Q. *et al.* RACK1 antagonizes TNF-α-induced cell death by promoting p38 activation. *Sci. Rep.* **5**, 14298; doi: 10.1038/srep14298 (2015).
